# Bosworth fracture complicated by unrecognized compartment syndrome: a case report and review of the literature

**DOI:** 10.1007/s00402-021-03815-1

**Published:** 2021-02-17

**Authors:** Jan Bartoníček, Stefan Rammelt, Karel Kostlivý

**Affiliations:** 1 Department of Orthopaedics, First Faculty of Medicine, Charles University and Central Military Hospital, U Vojenské Nemocnice 1200, Prague 6, 169 02 Czech Republic; 2 University Center of Orthopaedics, Trauma and Plastic Surgery, University Hospital Carl Gustav Carus Dresden, Fetscherstrasse 74, 01307 Dresden, Germany; 3 Department of Surgery of 1st Faculty of Medicine, Charles University and Thomayer Hospital, Videnska 800, Prague 4, 140 00 Czech Republic

**Keywords:** Ankle, Fracture, Locked dislocation, Compartment syndrome, Fasciotomy

## Abstract

**Introduction:**

Compartment syndrome (CS) is exceedingly rare in ankle fractures. However, the risk of CS development seems to be increased in the presence of a Bosworth fracture-dislocation (BF), a rare variant of locked dislocation of the fibula behind the tibia.

**Materials and methods:**

Here, we report the case of a 39-year old man with delayed diagnosis of CS after having sustained a BF and failed attempts on closed reduction. The patient developed a flexion contracture of the hallux necessitating secondary fusion.

**Results:**

At 3 years after the injury, the patient was capable of running, but had 10 degrees limitation of ankle dorsiflexion, persisting decreased sensation on the plantar surface and clawing of the lesser toes. A thorough review of the literature revealed nine cases of CS after 167 reported BF resulting in a calculated prevalence of 5.4%.

**Conclusions:**

Given the extreme paucity of CS in malleolar fractures, CS in BF has a relatively high prevalence. Risk factors include severe dislocations, repeated attempts on closed reduction, and a long interval to definite surgery. A high index of suspicion is required because delayed diagnosis leads to lasting functional restrictions.

Bosworth fracture (BF) is a rare type of ankle fracture-dislocation, characterized by entrapment of a fibular fragment behind the posterior surface of the tibia [1]. In Weber type B fractures it is the proximal fragment, while in Weber type C it is the distal fragment that is entrapped [2]. Recent CT-based studies have shown that BF is a severe injury regularly associated with fractures of the posterior malleolus and fraught with multiple possible complications including posttraumatic arthritis [3, 4]. In addition, BF seem to be prone to serious acute soft tissue complications like compartment syndrome (CS), skin necrosis, ankle stiffness and neurovascular damage [5–11]. Acute CS complicating a BF has been described for the first time by Szalay and Roberts [5], in 2001, and subsequently only by few other authors with delayed diagnosis in some cases [6–11].

The purpose of this study is to raise the awareness for this problem which may be neglected at first presentation and its implications on the basis of the authors´ own case and a thorough review of the literature. Over several years, we performed a literature review on Bosworth fracture-dislocations without time or language restriction [2–4]. For systematic research we used different combinations of the search terms “ankle” OR “malleolar” AND “fracture” OR “dislocation” AND “Bosworth” OR “locked dislocation” in PubMed, Medline, Google, Google Scholar, Scopus and selected textbooks until August 2020. Then, all articles pertaining to Bosworth fracture-dislocations were screened in the full text for the mention of a manifest compartment syndrome, symptoms of an impending compartment syndrome or late sequelae of a compartment syndrome.

## Case report

**Fig. 1:**
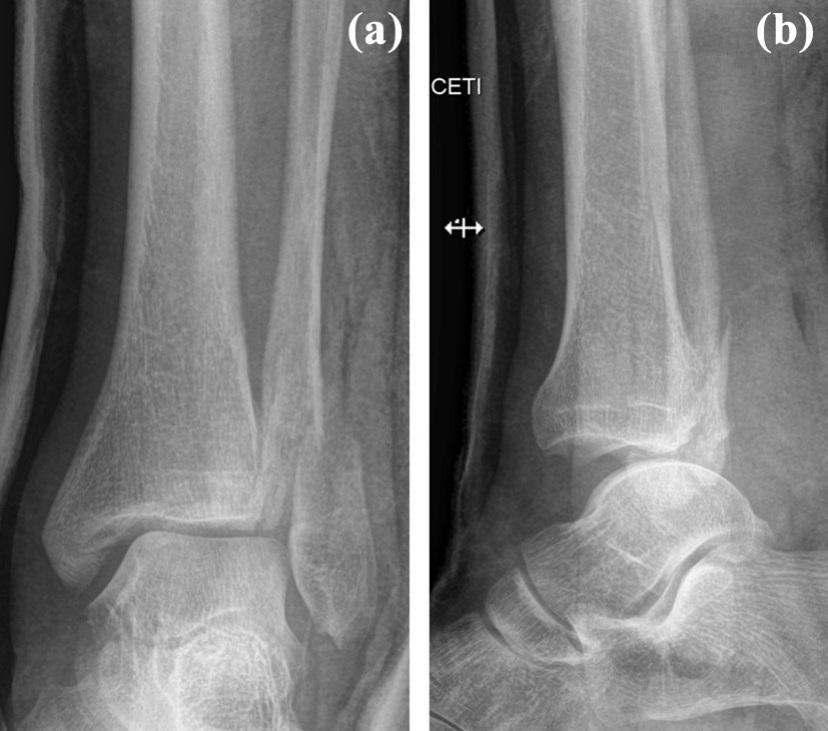
Initial anteroposterior and lateral radiographs with typical signs of a Bosworth fracture-dislocation: **a** overlap of the distal tibia and fibula, and **b** posterior dislocation of the talus and tibiofibular dissociation with the fibula being displaced posteriorly

A 39-year-old man slipped while running in the woods and severely twisted his left ankle. Immediately after the injury he experienced sharp pain in his left foot and ankle and was unable to walk. He was taken to a hospital and diagnosed with a Bosworth fracture of the left ankle 3 h after the injury. Radiographs showed a Weber type B fracture of the fibula, with a widened medial clear space and a concomitant fracture of the posterior malleolus. The presence of a Bosworth fracture-dislocation was suggested by overlap of the tibia and the proximal fragment of the fibula in the anteroposterior view, posterior subluxation of the talus and tibiofibular dissociation in the lateral view (Fig. 1).

**Fig. 2:**
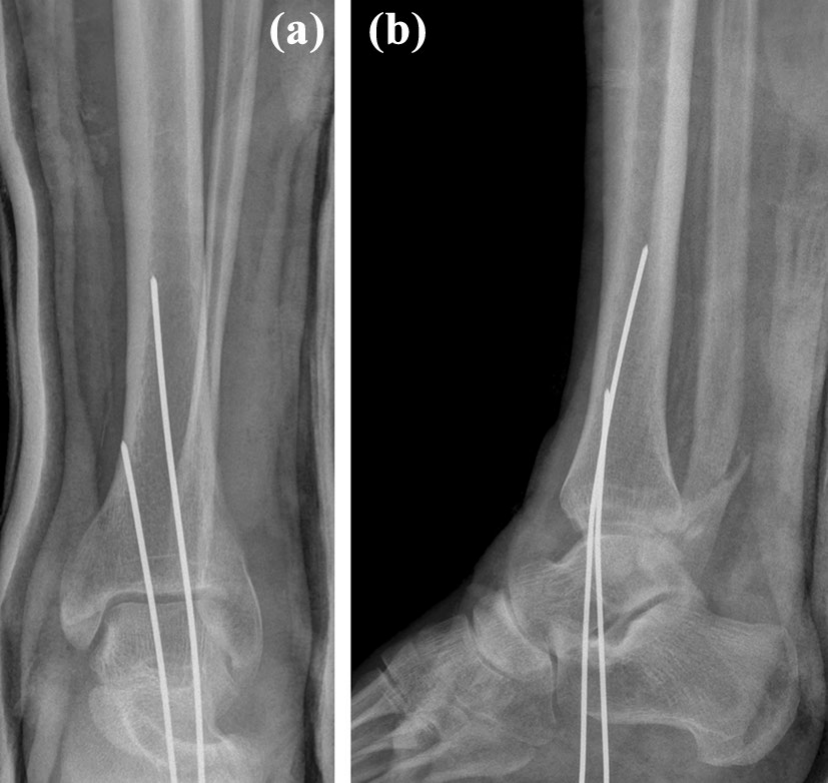
Radiograph after unsuccessful closed reduction under general anesthesia showing persisting **a** overlap of the distal tibia and fibula, and **b** tibiofibular dissociation

**Fig. 3:**
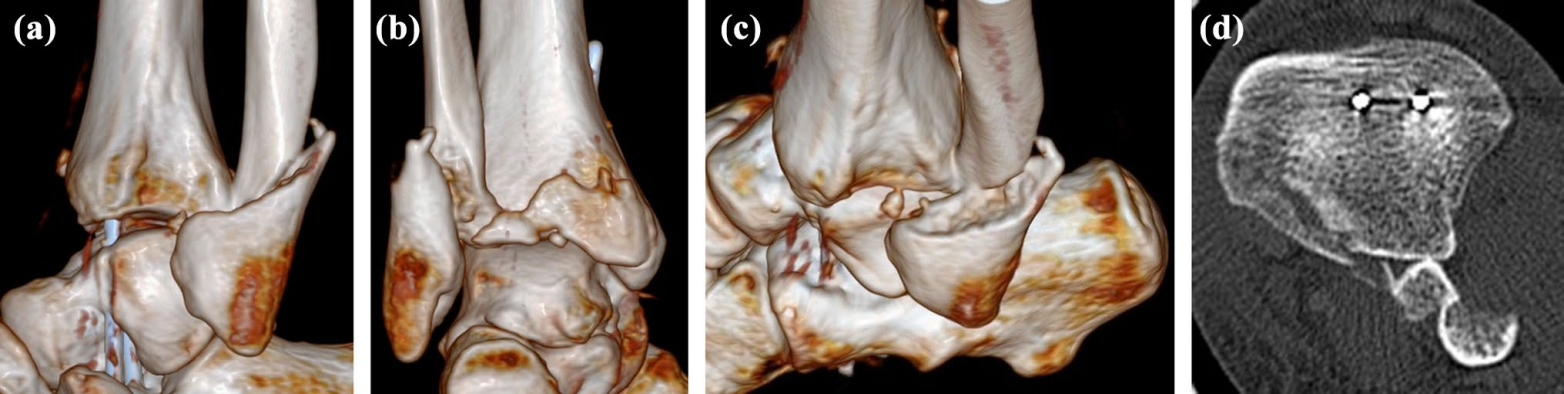
CT imaging demonstrated posterior displacement of the proximal fibular fragment fracture behind the distal tibia and a minimally displaced fracture of the posterior malleolus (Bartoníček-Rammelt type 3, two-part fracture with extension into the medial malleolus). **a** lateral 3D CT view; **b** posterior 3D CT view; **c** lateral 3D CT view; **d** axial CT scan

The following attempt at closed reduction under intravenous analgesia failed. The patient was admitted to the hospital and while waiting for reduction under general anesthesia the pain and swelling in his left foot increased and he began to develop paresthesia in the plantar aspect of the left forefoot. Closed reduction under general anesthesia was performed at the night of admission, 8 h after the injury. According to the treating surgeon, reduction was successful (Fig. 2). Following reduction, the pain subsided as a result of administration of analgesics. On the next morning, 15 h after the injury, the patient underwent CT examination, including 3D reconstructions. Although the results confirmed a proper diagnosis of a BF, they also showed failure of reduction. A posterior malleolus (PM) fracture representing type 3 of the Bartoníček-Rammelt classification [12] was detected. The proximal fragment of the fibula was entrapped behind the nondisplaced posterolateral part of the PM fragment (Fig. 3).

Swelling of the lower leg and the foot began to increase over time and blisters appeared around the ankle. A repeat reduction was performed 21 h after the injury under general anesthesia in the operating room with a bone hook introduced via a short incision over the lateral aspect of distal fibula. Reduction was successful and the ankle joint was then transfixed by 2 Kirschner wires.

**Fig. 4:**
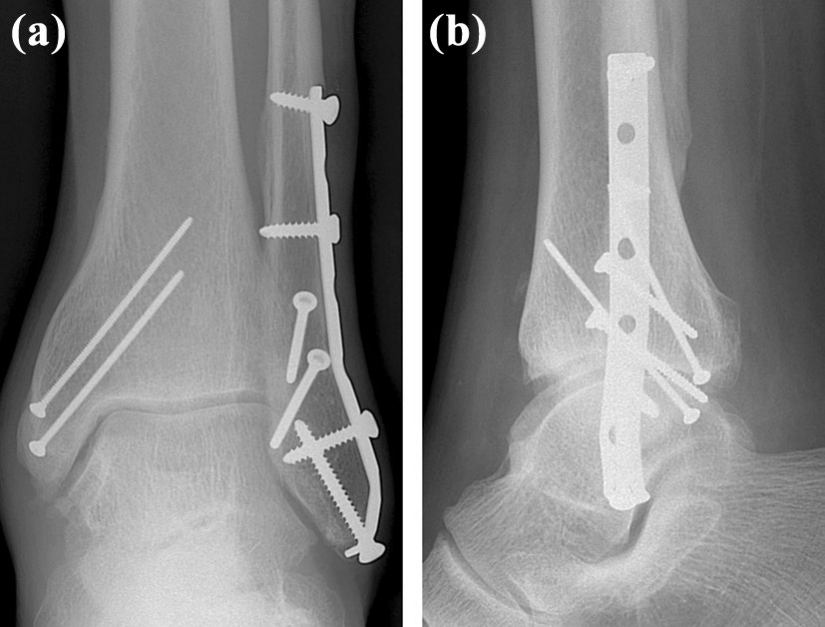
Postoperative radiographs after open reduction and internal fixation

The patient was admitted to the hospital. The involved lower limb was elevated and analgesics were administered. Pain, swelling and paresthesia slightly subsided. The condition of soft tissues allowed definite operative treatment 13 days after the injury. First, reduction of the posteromedial fragment of the PM and fixation with 2 pins were performed from a posteromedial approach. In the next step, the distal fibular fracture was reduced via a lateral approach and fixed with two 2.7 mm lag screws and a 3.5 mm 1/3 tubular neutralization plate. The hook test performed at this stage showed instability of the tibiofibular mortise that was subsequently fixed by a 3.5 mm tibiofibular syndesmotic screw (Fig. 4).

**Fig. 5:**
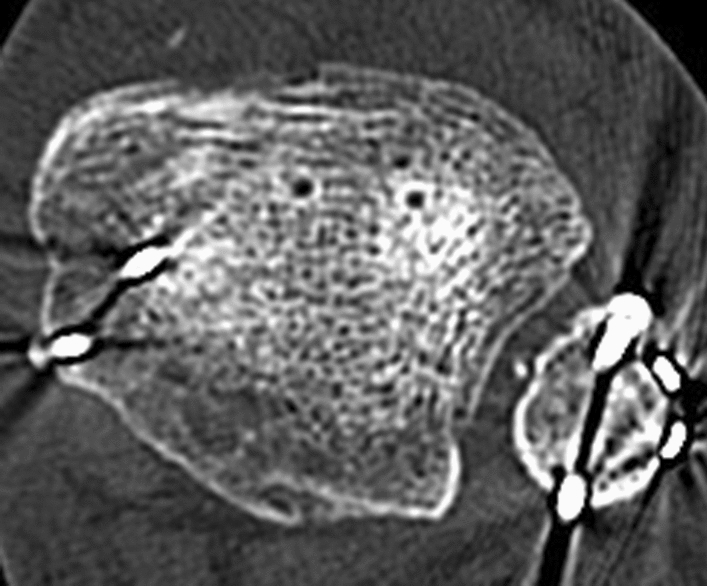
Postoperative CT control. **a** axial scan; **b** frontal scan

A split lower leg cast was applied for 6 weeks postoperatively. The surgical wounds healed uneventfully. In the postoperative period, the patient showed progressive paresthesia, mainly at the plantar surface of the foot, persisting swelling of the left ankle and development of a clawing of all toes, including a cockup deformity in the great toe. Sensation was diminished in the toes and almost absent in the area of the great toe and the first interdigital web space. The follow-up CT scan 2 months postoperatively documented a nearly anatomic reduction of all fragments, including the distal fibula into the fibular notch (Fig. 5). At that time, the syndesmotic screw was removed.

**Fig. 6:**
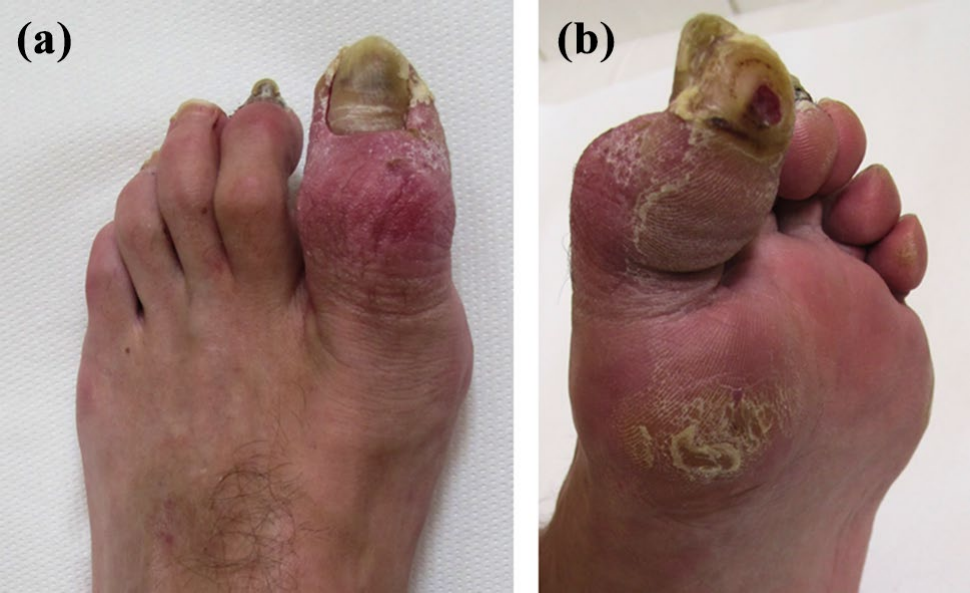
Clinical image of the formerly injured foot 3 months after surgery demonstrating typical lesions after compartment syndrome

At 3-month follow-up, a fixed flexion contracture of the great toe was observed without the possibility of passive correction, with ulcerations on the plantar and medial aspect of the great toe and below the first metatarsal head (Fig. 6). A follow-up radiograph proved healing of the fibular and PM fractures. Treatment of the ulceration and rehabilitation lasted for 6 months. Due to the persisting flexion contracture, a corrective fusion of the interphalangeal joint of the hallux was performed and fixed with two K-wires 18 months after the injury. K-wires were removed after solid bone union, 2 months postoperatively.

At the latest follow-up, 3 years after the injury, the patient was capable of full weight-bearing and jogging. Ankle plantarflexion was normal but dorsiflexion was limited by 10 degrees. The patient complained of persisting decreased sensation on the plantar surface and clawing of the 2^nd^ to 5^th^ toes of the involved foot. The great toe was fused in a plantigrade position and pain-free.

## Discussion and literature review

Compartment syndrome (CS) is an exceedingly rare complication after malleolar fractures typically affecting the deep posterior compartment of the lower leg [6, 13–15]. However, Bosworth fracture-dislocation (BF), a rare variant of an irreducible fracture-dislocation of the ankle, seems to be particularly prone to soft-tissue complications including CS.

The first to report on acute CS complicating a BF were Szalay and Roberts [5]. They described a case of a 28-year-old physician with a typical BF, who underwent three attempts at closed reduction. She was operated on 24 h after the injury. Approximately 8 h postoperatively, an anterior compartment syndrome of the lower leg began to develop. The patient underwent fasciotomy of all four compartments. Three years after surgery the patient had a mild residual hyperextension deformity of the great toe.

Beekman and Watson [6] reported on a 24-year-old patient who was sent home after a failed closed reduction attempt of the BF despite substantial soft-tissue swelling and returned to the emergency room 12 h later with already decreased plantar sensation and pain on passive stretch of the great toe. Multiple stick measurements revealed a CS with pathologic pressures in 3 of the 4 lower leg compartments and emergency fasciotomy of all 4 compartments was carried out. Following open reduction and internal fixation, the patient underwent two more surgical revisions with debridement of necrotic muscle from the anterior, lateral and deep posterior compartments. At one year, the patient still displayed a mildly antalgic gait with decreased dorsiflexion and plantar flexion of the ankle, continuing weakness of the toe flexors and extensors, and residual mild paresthesia on the plantar surface of the foot.

Chung [7] described a typical Bosworth fracture, i.e., a Weber type B fibula fracture and rupture of the deltoid ligament, with an anterior CS of the lower leg developing postoperatively and mild symptoms persisting at 14 months.

The patient reported by Lieder [8] underwent two unsuccessful closed reductions in the emergency department. The foot was noted to be cool and pulseless. CT angiogram demonstrated tapering of the flow of the anterior tibial, posterior tibial and peroneal arteries at the distal third of the lower leg and no visualization of the dorsalis pedis or plantar arteries. After urgent surgery, the patient was found to have an intact dorsalis pedis artery and posterior Doppler signals. At 11 months postoperatively, the patient reported restoration of sensation in his foot, however, a flexion deformity of the great toe persisted. Cho et al. [9], in a group of 16 BFs, found one case with CS, but did not specify details. In their series, the authors found the time interval to surgery and the number of manual reduction attempts to be negatively correlated with functional outcome.

Ren et al. [10] described a case of a 19-year-old patient who suffered a trimalleolar fracture of the ankle with a severe fixed external rotation deformity of the foot and skin tenting. The pulses of the dorsalis pedis and posterior tibial arteries could not be detected. The first attempt on closed reduction in the emergency room was unsuccessful. Radiographs showed a Weber type B fracture of the ankle and a fracture of the medial malleolus. CT scan proved a fracture of PM (Bartoníček Rammelt type 3), displacement of the proximal fibular fragment between the posterior aspect of distal tibia and the displaced PM. Reduction and internal fixation of the fibula and PM were performed via a posterolateral approach. One day after the operation, the patient developed postischemic CS, requiring fasciotomy. Eighteen months after the injury the function of the ankle was restored to normal and the patient had no subjective complaints.

Won et al. [11] analyzed a group of 51 BFs, which they divided into two groups. Group A included 36 patients operated on 24 h and later post-injury and group B comprised 15 patients operated on within 24 h. In group A, the authors found 2 cases of CS, requiring fasciotomy. An impending CS with BF was described by Ellanti et al. [16] and Yeoh et al. [17]. The complication was avoided by a timely open reduction in both cases and delayed wound closure in one case [17].

Overall, including the case presented here, 9 cases of compartment syndrome [5–11] have been described in the literature (Table 1). This results in a calculated prevalence of 5.4% out of 167 reported Bosworth fractures (Weber B and C types) [3, 4, 9–11, 18, 19]. Given the extreme paucity of CS in malleolar fractures, this is a high prevalence.

**Table 1:** Summary of reported cases with Bosworth fracture-dislocation and compartment syndrome

First author (year of publication)	Patient age	Patient sex	Specific remarks on CS and treatment	Fasciotomy	Outcome
Szalai (2001)	28	f	3 attempts on closed reduction Anterior CS	Yes	Mild hyperextension of great toe at 3 years
Beekman (2003)	24	m	1 attempt at closed reduction CS of 3 of 4 lower leg compartments	Yes	Muscle weakness, restricted motion, plantar hypesthesia at 1 year
Chung (2004)	42	m	1 attempt at closed reduction Anterior CS after surgery	No	Mild restriction of motion, hypesthesia in the region of the deep peroneal nerve at 14 months
Lieder (2014)	30	m	Pulseless foot after 2 attempts on closed reduction	No	Flexion deformity of great toe at 11 months
Cho (2019)	N. R	N R	Average of 2.2 failed attempts on closed reduction in 7 patients	N. R	Poorer overall outcomes with repeated closed reduction attempts and delayed surgery
Ren (2019)	19	m	Pulseless foot after 1 attempt on closed reduction	Yes	Normal function at 18 months
Won (2019)	N. R	N. R	Surgery > 24 h after the injury, CS diagnosed 8 h after surgery	Yes	Poorer overall outcomes with delayed surgery
Won (2019)	N. R	N. R	Surgery > 24 h after the injury, CS diagnosed 8 h after surgery	Yes	Poorer overall outcomes with delayed surgery
Presented case	39	m	2 attempts on closed reduction, persistent swelling and progressive clawing of the toes after surgery	No	Limited ankle dorsiflexion, fused first toe, clawing of lesser toes, plantar hypesthesia at 3 years

*CS* compartment syndrome, *N. R.* not reported, *m *male, *f* female

Besides CS, several reports on BF mention circulatory deficits resulting from the marked dislocation of the ankle. Fahey et al. [20], in 1956, described cyanosis, numbness and decreased temperature in two patients treated operatively for BF after repeated attempts at closed reduction had failed. The cases of Lieder et al. [8] and Ren et al. [10] described above could be interpreted as a postischemic CS following a circulatory deficit of the lower leg [21]. Further soft tissue complications associated with BF include skin necrosis and ankle stiffness particularly after delayed reduction [2, 3, 9, 19, 22].

The presented cases share certain common features. The first of them is a marked foot deformity upon admission, i.e., severe external rotation of the foot and posterior dislocation of the talus to the tibia often associated with skin tenting and circulatory deficits [8, 10, 20]. In all cases described in detail, there were repeated unsuccessful attempts at closed reduction that adds to the soft tissue damage [5–8, 20].

Another important prognostic factor is the interval between the injury and a successful (open) reduction. Significantly inferior results have been reported if reduction was delayed for more than 24 h [5, 6, 11]. Persistent dislocation increases the strain on the soft tissues thus potentially increasing the risk for soft tissue-related complications like skin necrosis, neurovascular damage, and CS. However, CS may also develop after a successful reduction [7, 8]. Furthermore, postischemic compartment syndrome may develop after successful revascularization [21] or restoring circulation through reduction as in two of the reported cases [8, 10].

In our case, the diagnosis of a BF was established properly, but the risk of CS development was underestimated. Furthermore, the quality of reduction performed under general anesthesia in the operating theatre was poor. In the further course, the development of CS was neglected, when analgesics partly obscured the typical pain. Diagnosis of a CS is primarily established clinically. The importance of clinical signs like increasing swelling and pain despite rest, cooling, elevation and pain medication, loss of skin wrinkling, blister formation, and pain on passive toe movement cannot be overestimated. Because the deep posterior compartment of the lower leg has a direct connection to the deep central (calcaneal) compartment of the foot, concurrent CS of the lower leg and foot also has to be considered [23, 24].

Early fasciotomy is warranted to avoid the late sequelae of CS of the lower leg and foot like rigid claw or hammertoe formation, cavus foot, equinovarus contracture, chronic pain or dysesthesia and callus or ulcer formation at the foot [21, 23, 24]. Some of these sequelae have been reported in the present case as well in the literature [5, 6, 8]. These deformities may warrant secondary corrective surgery and persistent functional deficits are likely to develop as muscle contracture resulting from CS is irreversible [21, 25, 26].

## Conclusions

While compartment syndrome is exceedingly rare after malleolar fractures it appears to be a relatively common complication of the Bosworth fracture-dislocation. This complication should be taken into account already during the initial examination of the patient. The risk of CS is increased with extreme external rotation of the foot, repeated attempts on closed reduction and delayed successful reduction of the ankle. The typical signs include massive swelling of the lower leg (and foot), blister formation, increasing pain despite rest, cooling and elevation, with paresthesia or loss of sensation already being a late sign. Radiographs show tibiofibular overlap in the anteroposterior view and tibiofibular diastasis and posterior displacement of the talus in lateral view. CT imaging should be performed in all suspected BFs as they will regularly reveal concomitant injuries like posterior malleolar fractures.

After confirmation of the diagnosis of a BF, it is essential to perform reduction as soon as possible. Closed reduction is almost always unsuccessful and repeated attempts will inevitably increase the soft tissue damage. Reduction must therefore always be performed under general or spinal anesthesia in an operating theatre. If immediate open reduction and internal fixation is impossible due to poor condition of the soft tissues or critical overall status of the patient, it is necessary to perform reduction of the displaced fibula percutaneously with a bone hook from a short incision above the fibula. Dermatofasciotomy must be performed if marked soft tissue swelling of the lower leg persists after successful reduction. The risk of a postischemic CS should be considered after a successful reduction and restoration of an initially compromised circulation. Therefore, continued peripheral anesthesia is contraindicated in these patients. Delayed recognition of CS regularly leads to persistent functional limitations.
